# Characterizing Longitudinal Patterns in Cognition, Mood, And Activity in Depression With 6-Week High-Frequency Wearable Assessment: Observational Study

**DOI:** 10.2196/46895

**Published:** 2024-05-31

**Authors:** Francesca Cormack, Maggie McCue, Caroline Skirrow, Nathan Cashdollar, Nick Taptiklis, Tempest van Schaik, Ben Fehnert, James King, Lambros Chrones, Sara Sarkey, Jasmin Kroll, Jennifer H Barnett

**Affiliations:** 1 Cambridge Cognition Cambridge United Kingdom; 2 Department of Psychiatry University of Cambridge Cambridge United Kingdom; 3 Cognition Kit Cambridge United Kingdom; 4 Takeda Pharmaceuticals USA Inc Lexington, MA United States; 5 Department of Psychological Science University of Bristol Bristol United Kingdom; 6 Ctrl Group London United Kingdom; 7 Fora Health London United Kingdom

**Keywords:** cognition, depression, digital biomarkers, ecological momentary assessment, mobile health, remote testing

## Abstract

**Background:**

Cognitive symptoms are an underrecognized aspect of depression that are often untreated. High-frequency cognitive assessment holds promise for improving disease and treatment monitoring. Although we have previously found it feasible to remotely assess cognition and mood in this capacity, further work is needed to ascertain the optimal methodology to implement and synthesize these techniques.

**Objective:**

The objective of this study was to examine (1) longitudinal changes in mood, cognition, activity levels, and heart rate over 6 weeks; (2) diurnal and weekday-related changes; and (3) co-occurrence of fluctuations between mood, cognitive function, and activity.

**Methods:**

A total of 30 adults with current mild-moderate depression stabilized on antidepressant monotherapy responded to testing delivered through an Apple Watch (Apple Inc) for 6 weeks. Outcome measures included cognitive function, assessed with 3 brief n-back tasks daily; self-reported depressed mood, assessed once daily; daily total step count; and average heart rate. Change over a 6-week duration, diurnal and day-of-week variations, and covariation between outcome measures were examined using nonlinear and multilevel models.

**Results:**

Participants showed initial improvement in the Cognition Kit N-Back performance, followed by a learning plateau. Performance reached 90% of individual learning levels on average 10 days after study onset. N-back performance was typically better earlier and later in the day, and step counts were lower at the beginning and end of each week. Higher step counts overall were associated with faster n-back learning, and an increased daily step count was associated with better mood on the same (*P*<.001) and following day (*P*=.02). Daily n-back performance covaried with self-reported mood after participants reached their learning plateau (*P*=.01).

**Conclusions:**

The current results support the feasibility and sensitivity of high-frequency cognitive assessments for disease and treatment monitoring in patients with depression. Methods to model the individual plateau in task learning can be used as a sensitive approach to better characterize changes in behavior and improve the clinical relevance of cognitive data. Wearable technology allows assessment of activity levels, which may influence both cognition and mood.

## Introduction

### Background

Major depressive disorder (MDD) is a debilitating condition and the leading cause of disease burden worldwide [1,2]. MDD is characterized primarily by low mood, or reduced interest and pleasure in daily activities [3]. Cognitive deficits are a substantial problem in patients with MDD, with reported impairments in a range of domains, including processing speed, attention, executive function, learning, and memory [4-6]. Despite impaired cognition being widely reported, further studies using objective neuropsychological measures are required [7].

### Self-Assessment of MDD

MDD is typically assessed using retrospective self-report, where patients reflect on their experiences over a period of days or weeks. However, this method of reporting is subject to a variety of memory distortions [8,9], and depression itself is linked to impaired recollection, memory bias, and overgeneralization [10]. A direct comparison between retrospective recall and repeated real-time assessments has shown negative emotional biases in patients with depression [11,12] which persist beyond the depressive episode in a subset of patients [13]. Various explanatory factors have been proposed, such as the number of previous episodes and demographic factors, but overall results have been inconsistent [14].

If patients’ recollections correspond poorly with their actual experiences, this is likely to distort our understanding of disease course and treatment response [15]. Discrepancies between objectively measured cognitive function and patients’ self-report have been demonstrated, with the latter being influenced by depressed mood [16-18]. This may be true even in cases of significant cognitive impairment, where self-reported cognitive function is associated with subjective complaints of depressive symptoms but not objective cognitive outcomes [19]. Hence, it is plausible that self-reported and objective assessments are measuring different cognitive capabilities, with objective measures needed to reveal underlying cognitive function.

Results from cognitive tests may also vary in relation to within-individual differences, including dietary effects and sleep-wake cycles [20,21]. These variances can make it difficult to differentiate clinically meaningful change from measurement error [22]. Higher frequency sampling is thought to generate more stable and reliable estimates of constructs of interest [22], by reducing state effects on punctual relationships, which can obscure the signal in study interventions, and by narrowing the margin of error [23].

### Real-Time Measurement of Cognition, Mood, and Activity

Advances in portable technology have enabled the precise, unobtrusive recording of real-time psychological, behavioral, and physiological measures. Higher frequency assessments in the context of everyday life can identify real life changes that are associated with clinical improvement [24], improve sensitivity for detecting change [25] and help to identify shifts in depressive symptoms [26]. Additionally, this approach allows for the characterization of the temporal relationship of symptoms over time in relation to changes in an individual’s behavior and environmental influences [27,28] creating a profile of the dynamic relationships between cognitive function, psychological processes, and biological processes of an individual [29].

However, this approach also has important implications for sampling strategy and data analysis. Diurnal changes in mood and affect have been reported in individuals with depression, where negative symptoms are more prevalent in the morning [30] and increased positive affect is seen later in the day [31,32]. Further, research indicates cognitive function follows the same pattern as daily changes in mood, with worse performance in the morning and better performance in the evening on a range of cognitive measures, including memory, attention, and psychomotor speed [33]. In the general population, more positive and less negative moods are typically seen on weekends, with improvements starting on Fridays [34], while more activity, measured by higher step counts, is noted during weekdays in comparison with weekends [35,36]. To account for the complexity of these fluctuations and how patients themselves may experience depression, novel data collection methods, such as ecological momentary assessments, are a promising opportunity to address this gap. Current scientific literature is mostly laboratory-based. Although ecological momentary assessment studies have shed light on various aspects of the disease, such as rumination and emotion reactivity [37], however, less is known about the relationship between cognition function and mood in MDD.

### Goal of This Study

Here we examine data from a 6-week feasibility study of daily cognitive, mood, and activity assessment in adults with mild-to-moderate depression stabilized on antidepressant treatment. Previously, in this same sample, we demonstrated excellent compliance with daily cognitive and mood assessments on a smart watch and good agreement with validated full-length cognitive and self-report measures [38]. High adherence rates were found (95%) with no deterioration over the course of the study. Adherence was not associated with depressive symptoms or cognitive functioning.

In this study, we aim to optimize methods for analyzing high-frequency longitudinal data and characterize relationships between cognition, mood, and activity data in order to facilitate future intervention studies. This study aims to examine (1) longitudinal changes in mood, cognition, activity levels, and heart rate over 6 weeks; (2) diurnal and weekday-related changes; and (3) the cooccurrence of fluctuations between mood, cognitive function, and activity.

## Methods

This was an observational study aimed at characterizing longitudinal patterns in cognition, mood, and activity in patients with MDD over 6 weeks with high-frequency assessment enabled by a wearable device.

### Participants

Full recruitment details have previously been reported by Cormack et al [38], who demonstrated the feasibility of high-frequency testing for a prolonged period of 6 weeks in participants with depression. In brief, participants were recruited for a primary psychiatric diagnosis of MDD with mild to moderate depression (as defined by Patient Health Questionnaire [PHQ]-9 scores between 5 and 15, inclusive). They were recruited through a patient recruitment company with links to primary care providers and patients with depression groups. Participants aged between 18 and 65 years, inclusive, who were able to read and understand English, were eligible for participation. Individuals were excluded if they had a personal history of another psychiatric disorder (except nonprimary anxiety); mental, neurological, or neurodegenerative disorders; or substance abuse or dependence. In total, 30 participants with MDD receiving antidepressant monotherapy treatment were enrolled.

### Measures

Cognitive function and mood were assessed with the Cognition Kit app (joint venture between Ctrl Group and Cambridge Cognition), loaded onto the Apple Watch (high-resolution touch-screen watch), and paired with an iPhone. Participants were asked to wear study equipment between 8 AM and 10 PM for 6 weeks. This duration was chosen as it is the time it typically takes for the improvement in mood to be seen following the start of administration of the antidepressant medication.

Prompts for cognitive assessment were given 3 times daily (morning, afternoon, and evening). Cognitive function was assessed with the Cognition Kit N-Back test, which has shown sensitivity to impairments in MDD [39]. Research suggests that n-back task performance is a marker of cognitive function, including aspects of working memory, task switching, and attention [40]. During test administration, 9 symbols randomly selected from a pool of 227 were presented briefly, one at a time, over 30 trials. Participants were asked to respond when any symbol was the same as the one presented 2 trials previously. The primary outcome measure was d-prime (the ratio of hits [correct detection of an n-back match] to false alarms [response during no match]). Under the current implementation of the paradigm, values of d-prime range from –3.33 to 3.33. Test-retest reliability of high frequency testing using n-back d-prime is 0.8 in a mixed sample of participants with neurodegenerative disease, immune-mediated inflammatory disorders, and healthy controls [41]. Each assessment took 30 seconds to complete, after which participants were shown their test score.

Prompts for mood assessment were given up to twice daily (afternoon and evening), with no prompt delivered in the evening if participants had completed the assessment in the afternoon. Mood was assessed with the following three questions adapted from the PHQ-2 and Perceived Deficits Questionnaire: How much have the following problems bothered you over the past day? (1) Lack of interest or pleasure in doing things; (2) Feeling down, depressed, or hopeless; and (3) Trouble concentrating on things (eg, newspapers or television). Responses were coded on a 4-point scale of severity of symptoms (1=no problem to 4=greatly). Responses were summated to provide a summary mood score. The PHQ-2, a short, validated form of the PHQ-9, has high accuracy and sensitivity to depression [42].

Total daily step count and average heart rate, measures associated with depressive symptom severity, [43,44], were acquired passively through the Apple Watch. The Apple Watch has been found to be a reliable and valid tool for assessing heart rate variability and is extremely accurate as a daily step count [45,46]. Similarity the n-back test has been previously used in wearable technology studies that incorporated high-frequency testing, demonstrating its feasibility [38,47].

### Procedure

Participants attended the study site, where researchers introduced the devices and tasks to participants, who were given the opportunity to practice using the software and hardware and ask questions. Testing was completed in the following 6 weeks (42 days), with data uploaded to secure study servers when transfer through Wi-Fi or roaming was possible. The study was completed with a home visit at 6 weeks, during which study hardware was returned.

Daily assessments were completed as part of a larger test battery, including full-length rating scales, and cognitive tests, and a semistructured qualitative interview, as described previously [38].

### Statistical Analysis

#### Data Cleaning

Data were harmonized by defining the first study day as the first day in which participants responded to assessment prompts for cognitive and mood assessments. Earlier days, only passive data were excluded from analyses. For activity and heart rate measures, non-wearing days (defined as days with <100 recorded steps [48,49], or where no heart rate was recorded) were excluded from the analyses (25 observations out of 1160).

Response frequency within each 1-hour period collapsed across days (eg, 6 AM-6:59 AM, 7 AM-7:59 AM, and 8 AM-8:59 AM) was examined separately for mood and d-prime to identify periods with sparse data. One-hour periods with n<25 assessments overall were excluded. This left n-back data from 6 AM-11:59 PM (n=29 observations dropped) and mood data from 12 noon until 5:59 PM (n=98 observations dropped). For all included 1-hour periods, there were between 41-287 data points available (mean 182 mood, 180 n-back assessments per 1-hour period).

#### Change Over Time

Since outcome measures are sampled repeatedly, hierarchical models are required to account for observations nested within individuals [50]. Random effects of participants with random intercept and random slope were applied to all models to control for between-participant variability, allowing the intercept and regression coefficient to vary between participants [51]. Model parameters were estimated using maximum likelihood, and model fits were compared with model likelihood ratios. This allowed the direct comparison of models with different fixed-effect structures using chi-square analysis and the selection of the best-fitting model. Diagnostic tests included examination of the normality of residuals and their spread for each predictor variable.

Raw data were transformed using log and square root transformations into normally distributed data as appropriate. A series of longitudinal mixed-effects models examined change in each outcome measure over the 6-week duration of the study to identify time-related trends in outcome measures. Each outcome measure was designated as the response variable, and a fixed effect of time-on-task was specified. Intercept-only models and linear, quadratic, and cubic trends of time-on-task were examined. For unequally spaced timepoints (cognitive assessment or mood), a continuous autoregressive correlation structure was applied. For summated daily indices (step count or heart rate), a first-order autoregressive covariance structure was used.

#### Diurnal and Weekday Effects

Fixed effects of diurnal variation or weekday were examined by appending these as additional predictors (fixed effects) to the best-fitting models. The time of day was treated as a continuous variable. Weekday was dummy coded from 1 to 7 (1=Monday to 7=Sunday). As in previous work, the quadratic and linear fixed effects of time of day and day of week were examined [32]. For measures with variation both in time-of-day and day-of-week (d-prime and mood), models first examined the effects of time-of-day and then incorporated weekday effects.

#### Characterizing Individual N-Back Learning Curves

N-back learning curves were characterized following previously described methods [52]. This includes (1) the “starting point,” the level at which performance begins; (2) the “asymptote,” the theoretically best score achievable toward which task performance tends with unlimited assessments; (3) the “slope,” the rate at which learning occurs; and (4) the “learning rate,” how quickly a prespecified level of performance is reached.

Intercepts extracted for each participant from the best-fitting mixed model for change over time using the analysis steps described above were implemented as individual starting points. These were subtracted from d-prime at each assessment occasion to provide a baseline-adjusted d-prime. As in previous work, the data were smoothed [53] by applying a 3-assessment moving average to the data series, corresponding to the typical number of assessments per day.

For each individual participant, nonlinear regression was used to fit an inverse curve (Y=*a*–(*b*/X)), yielding personalized estimates for *a* (asymptote, the theoretically best score achievable: as X →∞, Y →*a*) and *b* (slope) for baseline adjusted d-prime (Y) over each consecutive n-back assessment (X). A 90% learning rate was defined as the number of trials required by each individual participant to reach 90% of their potential beyond their starting point (Y=0.9a when X=10*b/*a*) [52]. The stable maximum d-prime was calculated for participants by summating their asymptotes and intercepts.

Exploratory correlations of individual asymptotes and slopes were completed with summary measures from mood and activity assessments. The data were first examined for normality, and parametric and nonparametric correlations were completed as appropriate.

#### Covariation of Fluctuations With Mood

Fluctuations over time were quantified using random effects. Residuals derived from the best-fitting hierarchical models described above, regressing out significant effects of time-on-task (to control for practice effects), diurnal effects, or weekday effects as relevant to each outcome measure. Residuals reflect the deviation of each individual from their own slope at each moment in time (better or worse, or higher or lower), quantifying the difference between each observation and what would be statistically expected. Since all measures, with the exception of d-prime, reflected overall daily measures, mean daily residuals for d-prime were computed by averaging d-prime residuals for each day, providing a consistent time scale for covariation analyses. Hierarchical models were used to examine covariation between measures. All models specified a first-order autoregressive covariance structure and random intercepts. Diagnostic tests included examination of the normality of residuals and their spread for each predictor variable.

A step-by-step approach was taken to examine the covariation of mood with d-prime and other assessment domains. First, a mixed model was used to predict fluctuations in mood based on fluctuations within all other domains (fixed effects: d-prime, activity, and heart rate). Next, the direction of causality was examined using a time-lagged approach. Analyses examined whether the fluctuations in mood were predicted by fluctuations in the predictor variable on the previous or following day. Only outcome measures that were significant predictors in concurrent covariation analyses were taken forward and included in the lagged models.

Analyses were repeated after excluding the period during which 90% of n-back learning took place (computed from individual participant learning curves), with the exception of participants who showed no significant slope where all data were included. This helped to identify correlation between measures after the influences of task learning were minimized. Where significant associations with mean daily d-prime fluctuations were seen, analyses were repeated when constrained only to days in which 3 n-back assessments were available. This aimed to test whether covariation between d-prime and mood was affected by regression to the mean (where days with fewer n-back assessments are likely to show greater variability).

### Ethical Considerations

The study was reviewed and approved by the Proportionate Review Sub-Committee of the Wales Research Ethics Committee at Swansea University (17/WA/0042) and performed in accordance with the current version of the Declaration of Helsinki. All participants provided written informed consent before being enrolled.

## Results

### Sample Characteristics

The final sample (N=30) included 19 women and 11 men, aged between 19 and 63 years (mean 37.2, SD 10.4 years). All participants received antidepressant monotherapy and had been on their current medication for an average of 9.9 (SD 9.5 months; range 0.4-94.3). Current medications included selective serotonin reuptake inhibitors (n=20), serotonin and norepinephrine reuptake inhibitors (n=5), tricyclic antidepressants (n=4), and serotonin antagonists and reuptake inhibitors (n=1). The mean depression symptom severity as measured by the PHQ-9 was 9.1 (SD 3.1; range 5-15).

### Modelling Changes in Mood, Cognition, Activity Levels, and Heart Rate Over 6 Weeks

Model selection statistics and resultant model parameters are presented in Table S1 in Multimedia Appendix 1. A cubic trend provided the best fit for change in cognitive performance over 6 weeks. Similarly, a linear trend provided the best fit for within-subjects change in mood over 6 weeks, in which depressive symptoms showed a subtle reduction over the course of the study. Heart rate and activity levels showed no overall change over time.

**Figure 1:**
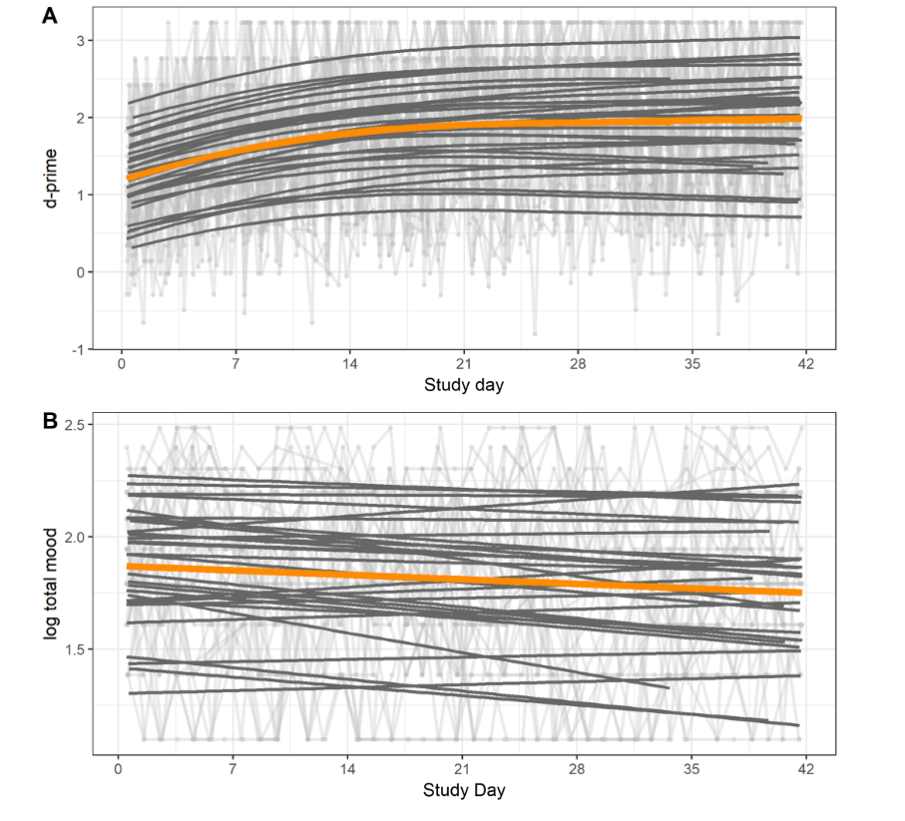
Change over study duration (A) in d-prime (up to 3 daily) is shown on the y-axis, with higher scores equating to better performance and (B) total mood as reflected by the y-axis (higher scores equate to a more depressed mood): random effects (bold gray lines), fixed effects (orange lines), model fits, and individual scores (pale gray lines) for individual participants.

Data for d-prime and mood are presented in Figure 1. Here, individual daily scores (light gray lines) are shown alongside fitted random effects (bold gray) and the fitted fixed effect (orange). This illustrates the variability across participants in the direction and magnitude of change over the 6 weeks and the remaining variability from these model fits.

### Diurnal and Weekday-Related Changes

**Figure 2:**
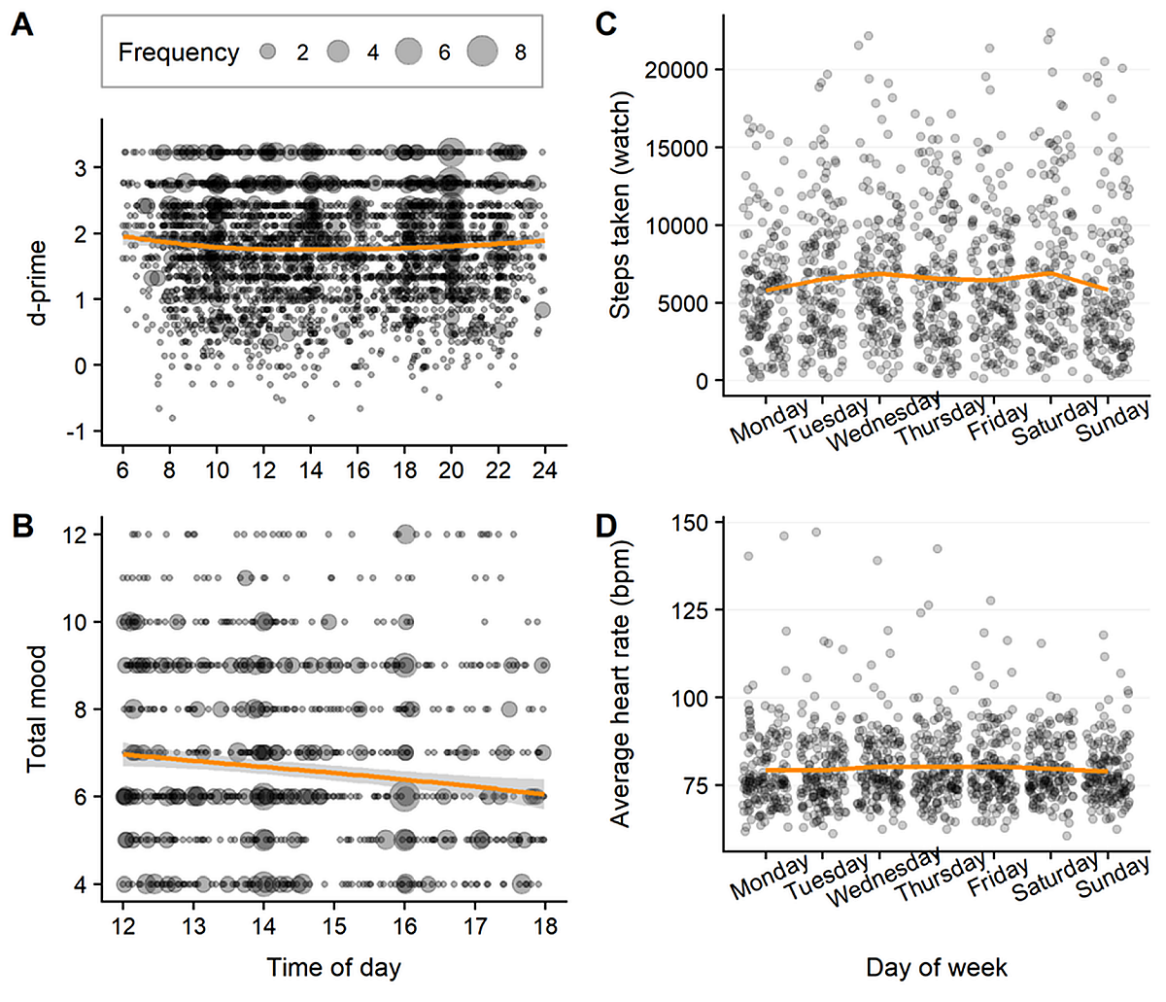
Diurnal and weekday effects in data: (A) Bubble chart of d-prime over time-of-day from early morning until midnight with loess regression line, with higher scores showing better performance; (B) bubble chart of total mood over time-of-day from noon until evening with loess regression line, with higher total mood denoting more severe depressive symptoms; (C) total steps taken over weekdays with mean line; and (D) average heart rate (beats per minute) over weekdays with mean line.

D-prime showed a significant quadratic effect of time-of-day, with better performance seen first thing in the morning and later at night (Figure 2A; time-of-day: estimate=–0.02, SE 0.01; *t*=–2.33; *P*=.02; time-of-day: estimate=0.001, SE 0.0006; *t*=2.48; *P*=.01). The model including time-of-day improved model fit (fit statistics: Akaike information criterion [AIC]=5952.15, Bayesian information criterion [BIC]=6019.10, likelihood ratio χ^2^=6.16; *P*=.01). No significant linear or quadratic effect of the weekday was observed (*P*≥.15).

Mood showed a subtle improvement over the course of the day, which did not reach significance thresholds and did not improve model fit (Figure 2B; time-of-day: estimate=–0.01, SE 0.006]; *P*=.06; model fit statistics: AIC=436.22, BIC=476.20, likelihood ratio=3.55; *P*=.06). The effects of day-of-week were also not significant (*P*≥.14).

Significant changes in activity and heart rate were also found over weekdays (Figure 2C-2D). Step counts were lower at the week onset and end (weekday: estimate=3.52, SE=1.50; *t*=3.52; *P*=.004; weekday: estimate=–0.64, SE 0.17; *t*=–3.66; *P*=.003). As expected, including these quadratic weekday effects improved model fit (fit statistics: AIC=10131.19, BIC=10166.41, likelihood ratio=–5058.60; *P*=.002). Similarly, heart rate showed a subtle quadratic effect of weekday with lower heart rates recorded on week onset and end (weekday: estimate=0.01, SE=0.006; *t*=1.96; *P*=.05, weekday: estimate=–0.002, SE=0.0008; *t*=–1.98; *P*=.05), with improved model fit (AIC=–2312.81, BIC=–2272.56, likelihood ratio=3.91; *P*=.05).

### Characterizing the N-Back Learning Curve

**Figure 3:**
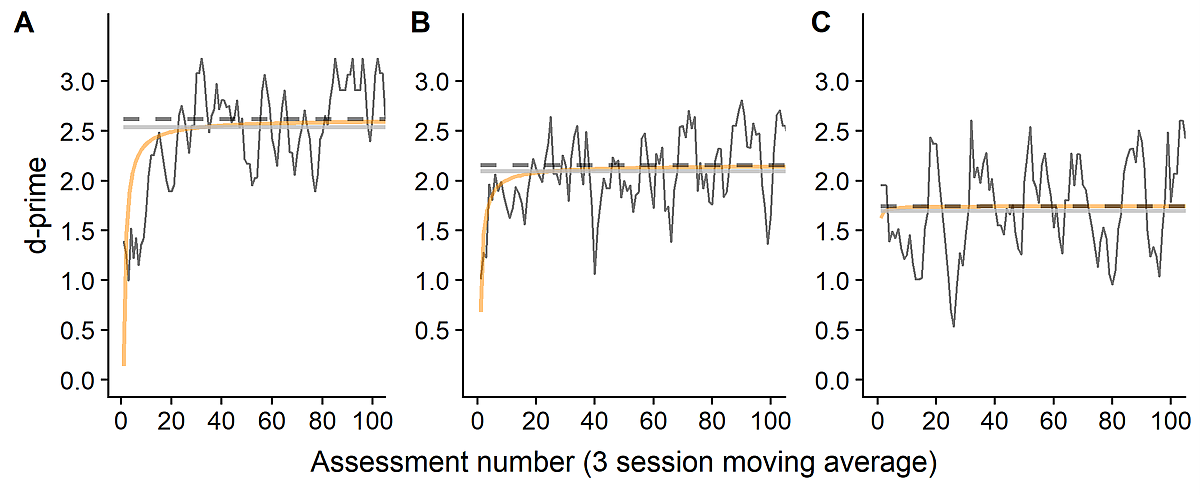
Examples of individual learning curves (dark gray lines), and inverse curves for n-back performance (orange line; y=asymptote–(slope/x)) over period of assessment. Asymptotes are shown in black dashed lines, while 90% learning rate is shown in pale gray. Subjects A (slope=2.48) and B (slope =1.47) show moderate and adequate fit for the inverse curve, while a poor fit is seen for subject C (slope=0.12).

An adequate fit of the inverse learning curve model was seen for 27 out of 30 participants of whom estimates for both slope and asymptote were significant (minimum *t*=2.18; *P*<.04). In participants with adequate fit, 90% learning rates were reached after an average of 22.4 assessments (range 13-31), occurring after a mean of 10 days (range 6-24). Participants with data showing poor fit displayed a flatter trajectory and lower slope over the period of assessment than those with a better fit (Figure 3). Stable maximal d-prime was seen at a mean of 1.94 (range 0.74-2.93), with 4 individuals having maximums within the top quintile of positive n-back scores (at 2.59 and above) and no participants with 95% CIs incorporating the highest possible score.

A total of 3 participants did not show a learning effect over the period of assessment, characterized by a nonsignificant slope. These participants performed overall above chance in n-back assessments (mean scores between 0.9 and 1.7), indicating adequate understanding of test objectives but no clear learning pattern.

Learning slope and asymptote correlated moderately with one another (ρ=0.70, 95% CI 0.39-0.84; *P*<.001), but neither slope nor asymptote correlated with the intercept (minimum *P*=.40). Correlational analysis examining the relationship between learning parameters and mean mood and activity during the monitoring period revealed no significant association between learning slope or asymptote with mean mood (ρ=–0.07 to 0.05; minimum *P*=.74), but a significant association emerged with mean activity (slope: ρ=0.44 [0.05-0.75]; *P*=.02; asymptote: ρ=0.45 [0.08-0.68]; *P*=.02).

### Covariation of Fluctuations Between Mood, Cognitive Function, and Activity

When examining the full assessment period, mood fluctuations did not significantly covary with fluctuations in daily heart rate (*t*=1.2; *P*=.23), or d-prime (*t*=–1.8; *P*=.07). However, daily mood fluctuations were seen with concurrent fluctuations in step count (estimate=–0.001, SE 0.0004; *t*=–3.93; *P*<.001), with a relatively higher levels of activity associated with relatively better mood. Examining the direction of these effects using lagged models for the entire assessment period revealed that increased step count the day before was associated with better mood on the following day (previous day activity residual: estimate=–0.001, SE 0.0004; *t*=–2.40; *P*=.02). However, a relatively more positive mood did not predict increased activity on the following day (*t*=–0.49; *P*=.62).

**Figure 4:**
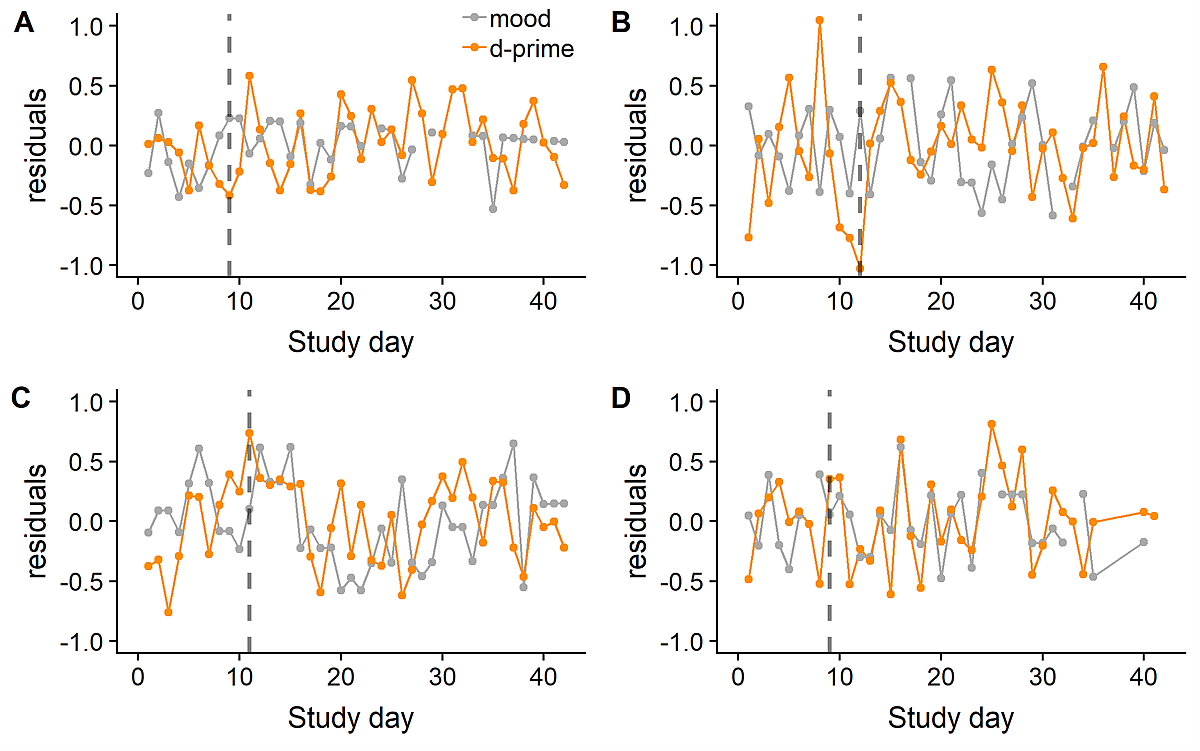
Covariation of daily mood residuals and mean daily d-prime residuals before and after 90% learning level reached (denoted in dashed line) for 4 participants. Note that mood residuals are inverted, so that higher residuals denote better outcomes for both mood and n-back performance.

Fluctuations between outcome variables and mood were reexamined after excluding the period during which 90% n-back learning rates were reached. Daily fluctuations in d-prime (estimate=–0.06, SE 0.02; *t*=–2.51; *P*=.01) and activity (estimate=–0.002, SE 0.0005; *t*=–3.50; *P*<.001) were associated with fluctuations in mood (Figure 4 shows individual examples of covariation of mood with d-prime). The covariation of mood with heart rate was nonsignificant (*t*=1.74; *P*=.08). Estimates and significance levels remained similar after restricting analyses to days where all 3 n-back assessments were available (d-prime: estimate=–0.07, SE 0.03; *t*=–2.57; *P*=.01; activity: estimate=–0.002, SE 0.0005; *t*=–4.42; *P*<.001). Taking a lagged approach, but after excluding the learning period, increased step count was again associated with better mood on the next day (estimate=–0.001, SE 0.0005; *t*=–2.00; *P*=.05). However, a relatively more positive mood did not predict an increased step count on the following day (*t*=–0.34; *P*=.73). This approach also indicated that relatively higher d-prime scores were not associated with better mood on the previous or following day (t range=0.61 to –0.77; *P* range=0.44 to 0.72).

## Discussion

### Principal Results and Comparison With Previous Work

This study characterizes high-frequency assessments of mood, cognition, and activity levels over a 6-week period in patients with MDD. By modelling individual learning curves from cognitive testing, we show that, after excluding an initial learning period, Cognition Kit N-Back test performance shows sensitivity to daily fluctuations in mood. The n-back test showed rapid early performance improvement followed by more incremental learning as participants neared a stable performance level, a trend that is replicated from previous findings for mean daily data in this sample [38]. This pattern is similar to that seen in laboratory-based cognitive assessments [53,54] and in practice effects identified during higher-frequency mobile cognitive assessments completed over 1 week of testing [22,55]. Participants completed 90% of task learning after a mean of 22 assessments (approximately 10 days after study onset). All participants achieved this level of improvement after 31 assessments (23 days). As such, a 10-day run-in before the introduction of any intervention may help to reduce the majority of learning effects in this n-back task, and subsequent performance can be referenced relative to an individual’s learning plateau as a proxy for baseline. Careful consideration of learning effects may be particularly important in intervention studies examining temporal associations between symptoms and accurate digital phenotyping [56]. As such, after the removal of learning effects, the association between mood and n-back performance can be objectively measured with a brief 3-times daily assessment. After excluding the initial learning period from the analysis, we found that better mood was associated with higher n-back scores on measurements taken on the same day. This approach demonstrates a potential method to “baseline” individual performance in high-frequency assessments after their learning plateau is reached in order to disentangle clinically meaningful relationships between mood and cognition. The absence of a significant relationship between mood and n-back performance during the learning period could be attributable to the development of specific learning strategies applied by participants during the learning phase [57,58] that obscure the relationship between mood and cognition.

Indeed, experimental studies have shown better cognitive performance when a positive mood is induced [59-61], although findings are not always consistent (eg, Nusbaum et al [62]). In the case of the latter study [62], this may be due to an examination of cognitive flexibility as opposed to working memory, as these may have distinct underlying mechanisms contributing toward overall cognitive performance [63]. Working memory impairments in patients with depression have been widely observed and are associated with the number of hospitalizations and the overall prognosis [64,65]. Performance in this domain is associated with negative symptom severity, including lack of motivation and apathy [66], in addition to positive valence [60]. In this study, changes may represent longer-term improvements in response to existing treatment regimens or may reflect the natural history of remission and fluctuating symptom levels commonly reported in depression [67]. It has been suggested that enhanced patient understanding of mood and mood changes over time may help to improve depressive symptoms and their management [68,69]. Whether these methods themselves have an impact on mood over time requires further clarification [70].

When examining diurnal and weekday effects, participants showed modestly better cognitive test performance early in the morning and late at night, with a slight decrease in function throughout the middle parts of the day. These findings are in agreement with the reported “afternoon slump” in cognition in healthy adults [71]. Neither diurnal nor day-of-week effects on mood were seen, in contrast to previous reports [31,34]. However, in broad agreement with previous research [35,36], we identified day-of-week effects for activity levels as measured by step count and similar patterns in mean heart rate. Step counts showed a quadratic effect of day of week, with lower counts registered at the beginning and end of each week. These findings highlight the importance of carefully considering the timing of assessments to ensure consistency in data sampling and control trends in data over time. Longer-term trends in mood data independent of any treatment effects highlight the importance of adequately controlling for subtle overall trends in time in future interventional studies, ideally using a randomized design and a placebo-controlled comparison group. Using a multilevel modeling approach, we were able to examine whether mood fluctuated synchronously with other measures of interest over time. We identified covariations in mood and activity levels, in keeping with a body of research showing improvements in depression symptoms with exercise [72,73], or simple walking interventions [74]. Our results show that a relatively increased mood was associated with a relatively increased step count on the previous day (but not the following day), which indicated that the beneficial effects of exercise on well-being may well be protracted [75].

### Limitations

While associations between activity levels (step count), mood, and cognition were identified, step counts are likely to have been influenced by wearing patterns [38], thereby reducing the reliability of the data. The summarized daily step counts also fail to elucidate whether activity was acute or prolonged, vigorous, or light, and the exact timing of activity changes in relation to mood and cognitive assessments. Accelerometers can help to continuously monitor activity, and concurrent GPS and travel diary information has been found to help to classify and identify the duration of walking [76]. Previous studies have examined associations between physical activity on mood in assessments triggered through GPS distance tracking [77,78], a method that could be used to refine the timing and accuracy of activity assessments in this context. Additionally, since this study focused on patients with mild-to-moderate MDD who volunteered for participation, it is unclear whether the results would generalize to patients with different clinical severity.

### Conclusions

This study indicates the importance of incorporating objective measures of cognitive testing and provides insight into fluctuations in mood and cognition in patients with MDD. The feasibility of remote high-frequency testing in MDD is promising for future research in this field and has important implications for clinical interventions. While these methods can be used to monitor nuanced fluctuations between mood and cognition in a real-life setting, they may also be useful as a treatment tool. Understanding objective cognitive function in depression may also be used to target patients’ acceptance of their objective difficulties in cognition and may be particularly relevant for interventions such as CBT and mindfulness [79]. Overall, while assessments of activity need to be further refined and improved, the effects of step count on mood and cognition support the concurrent capture of data on activity, which may be an important contributor to variations in mood and changes in cognition in everyday life in patients with depression.

## Appendix

Multimedia Appendix 1 Final model selection and model parameters for daily assessment outcomes over 6-week period.

## Abbreviations

AIC: Akaike information criterion
BIC: Bayesian information criterion
MDD: major depressive disorder
PHQ: Patient Health Questionnaire

## References

[1] Ferrari AJ, Charlson FJ, Norman RE, Patten SB, Freedman G, Murray CJL, Vos T, Whiteford HA (2013). Burden of depressive disorders by country, sex, age, and year: findings from the Global Burden of Disease Study 2010. PLoS Med.

[2] Moussavi S, Chatterji S, Verdes E, Tandon A, Patel V, Ustun B (2007). Depression, chronic diseases, and decrements in health: results from the World Health Surveys. Lancet.

[3] (2013). Diagnostic and Statistical Manual of Mental Disorders, 5th Edition.

[4] Hammar A, Ardal G (2009). Cognitive functioning in major depression—a summary. Front Hum Neurosci.

[5] McClintock SM, Husain MM, Greer TL, Cullum CM (2010). Association between depression severity and neurocognitive function in major depressive disorder: a review and synthesis. Neuropsychology.

[6] Ahern E, Semkovska M (2017). Cognitive functioning in the first-episode of major depressive disorder: a systematic review and meta-analysis. Neuropsychology.

[7] Kaser M, Zaman R, Sahakian BJ (2017). Cognition as a treatment target in depression. Psychol Med.

[8] Robinson MD, Clore GL (2002). Belief and feeling: evidence for an accessibility model of emotional self-report. Psychol Bull.

[9] Ebner-Priemer UW, Trull TJ (2009). Ambulatory assessment: an innovative and promising approach for clinical psychology. European Psychologist.

[10] Dillon DG, Pizzagalli DA (2018). Mechanisms of memory disruption in depression. Trends Neurosci.

[11] Ben-Zeev D, Young MA (2010). Accuracy of hospitalized depressed patients' and healthy controls' retrospective symptom reports: an experience sampling study. J Nerv Ment Dis.

[12] Ben-Zeev D, Young MA, Madsen JW (2009). Retrospective recall of affect in clinically depressed individuals and controls. Cogn Emot.

[13] Everaert J, Vrijsen JN, Martin-Willett R, van de Kraats L, Joormann J (2022). A meta-analytic review of the relationship between explicit memory bias and depression: depression features an explicit memory bias that persists beyond a depressive episode. Psychological Bulletin.

[14] Semkovska M, Quinlivan L, O'Grady T, Johnson R, Collins A, O'Connor J, Knittle H, Ahern E, Gload T (2019). Cognitive function following a major depressive episode: a systematic review and meta-analysis. Lancet Psychiatry.

[15] Bos FM, Schoevers RA, aan het Rot M (2015). Experience sampling and ecological momentary assessment studies in psychopharmacology: a systematic review. Eur Neuropsychopharmacol.

[16] Popkin SJ, Gallagher D, Thompson LW, Moore M (1982). Memory complaint and performance in normal and depressed older adults. Exp Aging Res.

[17] Antikainen R, Hänninen T, Honkalampi K, Hintikka J, Koivumaa-Honkanen H, Tanskanen A, Viinamäki H (2001). Mood improvement reduces memory complaints in depressed patients. Eur Arch Psychiatry Clin Neurosci.

[18] Naismith SL, Longley WA, Scott EM, Hickie IB (2007). Disability in major depression related to self-rated and objectively-measured cognitive deficits: a preliminary study. BMC Psychiatry.

[19] Zlatar ZZ, Muniz M, Galasko D, Salmon DP (2018). Subjective cognitive decline correlates with depression symptoms and not with concurrent objective cognition in a clinic-based sample of older adults. J Gerontol B Psychol Sci Soc Sci.

[20] Smith AP, Clark R, Gallagher J (1999). Breakfast cereal and caffeinated coffee: effects on working memory, attention, mood, and cardiovascular function. Physiol Behav.

[21] Wright KP, Lowry CA, Lebourgeois MK (2012). Circadian and wakefulness-sleep modulation of cognition in humans. Front Mol Neurosci.

[22] Schweitzer P, Husky M, Allard M, Amieva H, Pérès K, Foubert-Samier A, Dartigues JF, Swendsen J (2017). Feasibility and validity of mobile cognitive testing in the investigation of age-related cognitive decline. Int J Methods Psychiatr Res.

[23] Moore RC, Depp CA, Wetherell JL, Lenze EJ (2016). Ecological momentary assessment versus standard assessment instruments for measuring mindfulness, depressed mood, and anxiety among older adults. J Psychiatr Res.

[24] Barge-Schaapveld DQ, Nicolson NA, van der Hoop RG, De Vries MW (1995). Changes in daily life experience associated with clinical improvement in depression. J Affect Disord.

[25] Lenderking WR, Hu M, Tennen H, Cappelleri JC, Petrie CD, Rush AJ (2008). Daily process methodology for measuring earlier antidepressant response. Contemp Clin Trials.

[26] van de Leemput IA, Wichers M, Cramer AOJ, Borsboom D, Tuerlinckx F, Kuppens P, van Nes EH, Viechtbauer W, Giltay EJ, Aggen SH, Derom C, Jacobs N, Kendler KS, van der Maas HLJ, Neale MC, Peeters F, Thiery E, Zachar P, Scheffer M (2014). Critical slowing down as early warning for the onset and termination of depression. Proc Natl Acad Sci U S A.

[27] Trull TJ, Ebner-Priemer U (2013). Ambulatory assessment. Annu Rev Clin Psychol.

[28] Marzano L, Bardill A, Fields B, Herd K, Veale D, Grey N, Moran P (2015). The application of mHealth to mental health: opportunities and challenges. Lancet Psychiatry.

[29] Sliwinski MJ, Mogle JA, Hyun J, Munoz E, Smyth JM, Lipton RB (2018). Reliability and validity of ambulatory cognitive assessments. Assessment.

[30] Leibenluft E, Noonan BM, Wehr TA (1992). Diurnal variation: reliability of measurement and relationship to typical and atypical symptoms of depression. J Affect Disord.

[31] Peeters F, Berkhof J, Delespaul P, Rottenberg J, Nicolson NA (2006). Diurnal mood variation in major depressive disorder. Emotion.

[32] Crowe E, Daly M, Delaney L, Carroll S, Malone KM (2019). The intra-day dynamics of affect, self-esteem, tiredness, and suicidality in major depression. Psychiatry Res.

[33] Porterfield T, Cook M, Deary IJ, Ebmeier KP (1997). Neuropsychological function and diurnal variation in depression. J Clin Exp Neuropsychol.

[34] Stone AA, Schneider S, Harter JK (2012). Day-of-week mood patterns in the United States: on the existence of 'Blue Monday', 'Thank God it's Friday' and weekend effects. J Posit Psychol.

[35] Hirvensalo M, Telama R, Schmidt MD, Tammelin TH, Yang X, Magnussen CG, Vkari JS, Raitakari OT (2011). Daily steps among Finnish adults: variation by age, sex, and socioeconomic position. Scand J Public Health.

[36] Ludwig VM, Bayley A, Cook DG, Stahl D, Treasure JL, Asthworth M, Greenough A, Winkley K, Bornstein SR, Ismail K (2018). Association between depressive symptoms and objectively measured daily step count in individuals at high risk of cardiovascular disease in South London, UK: a cross-sectional study. BMJ Open.

[37] Colombo D, Fernández-Álvarez J, Patané A, Semonella M, Kwiatkowska M, García-Palacios A, Cipresso P, Riva G, Botella C (2019). Current state and future directions of technology-based ecological momentary assessment and intervention for major depressive disorder: a systematic review. J Clin Med.

[38] Cormack F, McCue M, Taptiklis N, Skirrow C, Glazer E, Panagopoulos E, van Schaik TA, Fehnert B, King J, Barnett JH (2019). Wearable technology for high-frequency cognitive and mood assessment in major depressive disorder: longitudinal observational study. JMIR Ment Health.

[39] Snyder HR (2013). Major depressive disorder is associated with broad impairments on neuropsychological measures of executive function: a meta-analysis and review. Psychol Bull.

[40] Espeland MA, Katula JA, Rushing J, Kramer AF, Jennings JM, Sink KM, Nadkarni NK, Reid KF, Castro CM, Church T, Kerwin DR, Williamson JD, Marottoli RA, Rushing S, Marsiske M, Rapp SR (2013). Performance of a computer-based assessment of cognitive function measures in two cohorts of seniors. Int J Geriatr Psychiatry.

[41] Cormack F, Ticcinelli V, Taptiklis N, Kudelka J, Emmert K, Maetzler W, Reilmann R, Latzman RD, Ng WF, McRae V, Davies K, van der Woude J, Ahmaniemi T, Chatterjee M, Fierrez J (2022). App-based cognitive assessment and monitoring—a feasibility study in patients with immune-mediated inflammatory and neurodegenerative disorders. Neurosci Appl.

[42] Levis B, Sun Y, He C, Wu Y, Krishnan A, Bhandari PM, Neupane D, Imran M, Brehaut E, Negeri Z, Fischer FH, Benedetti A, Thombs BD, Che L, Levis A, Riehm K, Saadat N, Azar M, Rice D, Boruff J, Kloda L, Cuijpers P, Gilbody S, Ioannidis J, McMillan D, Patten S, Shrier I, Ziegelstein R, Moore A, Akena D, Amtmann D, Arroll B, Ayalon L, Baradaran H, Beraldi A, Bernstein C, Bhana A, Bombardier C, Buji RI, Butterworth P, Carter G, Chagas M, Chan J, Chan LF, Chibanda D, Cholera R, Clover K, Conway A, Conwell Y, Daray F, de Man-van Ginkel J, Delgadillo J, Diez-Quevedo C, Fann J, Field S, Fisher J, Fung D, Garman E, Gelaye B, Gholizadeh L, Gibson L, Goodyear-Smith F, Green E, Greeno C, Hall B, Hampel P, Hantsoo L, Haroz E, Harter M, Hegerl U, Hides L, Hobfoll S, Honikman S, Hudson M, Hyphantis T, Inagaki M, Ismail K, Jeon HJ, Jetté N, Khamseh M, Kiely K, Kohler S, Kohrt B, Kwan Y, Lamers F, Lara MA, Levin-Aspenson H, Lino V, Liu SI, Lotrakul M, Loureiro S, Löwe B, Luitel N, Lund C, Marrie RA, Marsh L, Marx B, McGuire A, Sidik SM, Munhoz T, Muramatsu K, Nakku J, Navarrete L, Osório F, Patel V, Pence B, Persoons P, Petersen I, Picardi A, Pugh S, Quinn T, Rancans E, Rathod S, Reuter K, Roch S, Rooney A, Rowe H, Santos I, Schram M, Shaaban J, Shinn E, Sidebottom A, Simning A, Spangenberg L, Stafford L, Sung S, Suzuki K, Swartz R, Tan PLL, Taylor-Rowan M, Tran T, Turner A, van der Feltz-Cornelis C, van Heyningen T, van Weert H, Wagner L, Li Wang J, White J, Winkley K, Wynter K, Yamada M, Zeng QZ, Zhang Y (2020). Accuracy of the PHQ-2 alone and in combination with the PHQ-9 for screening to detect major depression: systematic review and meta-analysis. JAMA.

[43] Schiweck C, Piette D, Berckmans D, Claes S, Vrieze E (2019). Heart rate and high frequency heart rate variability during stress as biomarker for clinical depression. A systematic review. Psychol Med.

[44] Ramsey CM, Lynch KG, Gehrman PR, Vairavan S, Narayan VA, Li QS, Oslin DW (2022). Daily steps and depressive symptoms: a longitudinal evaluation of patients with major depressive disorder in the Precision Medicine in Mental Health Care study. J Affect Disord.

[45] Veerabhadrappa P, Moran MD, Renninger MD, Rhudy MB, Dreisbach SB, Gift KM (2018). Tracking steps on Apple Watch at different walking speeds. J Gen Intern Med.

[46] Fuller D, Colwell E, Low J, Orychock K, Tobin MA, Simango B, Buote R, Van Heerden D, Luan H, Cullen K, Slade L, Taylor NGA (2020). Reliability and validity of commercially available wearable devices for measuring steps, energy expenditure, and heart rate: systematic review. JMIR Mhealth Uhealth.

[47] Brose A, Schmiedek F, Lövdén M, Lindenberger U (2012). Daily variability in working memory is coupled with negative affect: the role of attention and motivation. Emotion.

[48] Richardson CR, Buis LR, Janney AW, Goodrich DE, Sen A, Hess ML, Mehari KS, Fortlage LA, Resnick PJ, Zikmund-Fisher BJ, Strecher VJ, Piette JD (2010). An online community improves adherence in an internet-mediated walking program. Part 1: results of a randomized controlled trial. J Med Internet Res.

[49] Moy ML, Weston NA, Wilson EJ, Hess ML, Richardson CR (2012). A pilot study of an internet walking program and pedometer in COPD. Respir Med.

[50] Bringmann LF, Vissers N, Wichers M, Geschwind N, Kuppens P, Peeters F, Borsboom D, Tuerlinckx F (2013). A network approach to psychopathology: new insights into clinical longitudinal data. PLoS One.

[51] Verhagen SJW, Hasmi L, Drukker M, van Os J, Delespaul PAEG (2016). Use of the experience sampling method in the context of clinical trials. Evid Based Ment Health.

[52] Feldman LS, Cao J, Andalib A, Fraser S, Fried GM (2009). A method to characterize the learning curve for performance of a fundamental laparoscopic simulator task: defining "learning plateau" and "learning rate". Surgery.

[53] Yamashita M, Kawato M, Imamizu H (2015). Predicting learning plateau of working memory from whole-brain intrinsic network connectivity patterns. Sci Rep.

[54] Collie A, Maruff P, Darby DG, McStephen M (2003). The effects of practice on the cognitive test performance of neurologically normal individuals assessed at brief test-retest intervals. J Int Neuropsychol Soc.

[55] Dupuy M, Misdrahi D, N'Kaoua B, Tessier A, Bouvard A, Schweitzer P, Auriacombe M, Serre F, Fatseas M, Swendsen J (2018). Mobile cognitive testing in patients with schizophrenia: a controlled study of feasibility and validity. J de Ther Comport et Cogn.

[56] Griffin B, Saunders KEA (2019). Smartphones and wearables as a method for understanding symptom mechanisms. Front Psychiatry.

[57] Gathercole SE, Dunning DL, Holmes J, Norris D (2016). Working memory training involves learning new skills. J Mem Lang.

[58] Tenison C, Anderson JR (2016). Modeling the distinct phases of skill acquisition. J Exp Psychol Learn Mem Cogn.

[59] Nadler RT, Rabi R, Minda JP (2010). Better mood and better performance. Learning rule-described categories is enhanced by positive mood. Psychol Sci.

[60] Storbeck J, Maswood R (2016). Happiness increases verbal and spatial working memory capacity where sadness does not: emotion, working memory and executive control. Cogn Emot.

[61] Yang H, Yang S, Isen AM (2013). Positive affect improves working memory: implications for controlled cognitive processing. Cogn Emot.

[62] Nusbaum AT, Wilson CG, Stenson A, Hinson JM, Whitney P (2018). Induced positive mood and cognitive flexibility: evidence from task switching and reversal learning. Collabra Psychol.

[63] Periáñez JA, Lubrini G, García-Gutiérrez A, Ríos-Lago M (2021). Construct validity of the Stroop color-word test: influence of speed of visual search, verbal fluency, working memory, cognitive flexibility, and conflict monitoring. Arch Clin Neuropsychol.

[64] Harvey PO, Le Bastard G, Pochon JB, Levy R, Allilaire JF, Dubois B, Fossati P (2004). Executive functions and updating of the contents of working memory in unipolar depression. J Psychiatr Res.

[65] Nikolin S, Tan YY, Schwaab A, Moffa A, Loo CK, Martin D (2021). An investigation of working memory deficits in depression using the n-back task: a systematic review and meta-analysis. J Affect Disord.

[66] Zhu Y, Womer FY, Leng H, Chang M, Yin Z, Wei Y, Zhou Q, Fu S, Deng X, Lv J, Song Y, Ma Y, Sun X, Bao J, Wei S, Jiang X, Tan S, Tang Y, Wang F (2019). The relationship between cognitive dysfunction and symptom dimensions across schizophrenia, bipolar disorder, and major depressive disorder. Front Psychiatry.

[67] Andrews G (2001). Placebo response in depression: bane of research, boon to therapy. Br J Psychiatry.

[68] Kauer SD, Reid SC, Crooke AHD, Khor A, Hearps SJC, Jorm AF, Sanci L, Patton G (2012). Self-monitoring using mobile phones in the early stages of adolescent depression: randomized controlled trial. J Med Internet Res.

[69] Groot PC (2010). Patients can diagnose too: how continuous self-assessment aids diagnosis of, and recovery from, depression. J Ment Health.

[70] Hartmann JA, Wichers M, Menne-Lothmann C, Kramer I, Viechtbauer W, Peeters F, Schruers KRJ, van Bemmel AL, Myin-Germeys I, Delespaul P, van Os J, Simons CJP (2015). Experience sampling-based personalized feedback and positive affect: a randomized controlled trial in depressed patients. PLoS One.

[71] Wertz AT, Ronda JM, Czeisler CA, Wright KP (2006). Effects of sleep inertia on cognition. JAMA.

[72] Schuch FB, Vancampfort D, Richards J, Rosenbaum S, Ward PB, Stubbs B (2016). Exercise as a treatment for depression: a meta-analysis adjusting for publication bias. J Psychiatr Res.

[73] Schuch FB, Deslandes AC, Stubbs B, Gosmann NP, da Silva CTB, de Almeida Fleck MP (2016). Neurobiological effects of exercise on major depressive disorder: a systematic review. Neurosci Biobehav Rev.

[74] Robertson R, Robertson A, Jepson R, Maxwell M (2012). Walking for depression or depressive symptoms: a systematic review and meta-analysis. Ment Health Phys Act.

[75] Daley AJ, Welch A (2004). The effects of 15 min and 30 min of exercise on affective responses both during and after exercise. J Sports Sci.

[76] Kang B, Moudon AV, Hurvitz PM, Reichley L, Saelens BE (2013). Walking objectively measured: classifying accelerometer data with GPS and travel diaries. Med Sci Sports Exerc.

[77] Reichert M, Tost H, Reinhard I, Schlotz W, Zipf A, Salize HJ, Meyer-Lindenberg A, Ebner-Priemer UW (2017). Exercise versus nonexercise activity: e-diaries unravel distinct effects on mood. Med Sci Sports Exerc.

[78] Reichert M, Tost H, Reinhard I, Zipf A, Salize HJ, Meyer-Lindenberg A, Ebner-Priemer UW (2016). Within-subject associations between mood dimensions and non-exercise activity: an ambulatory assessment approach using repeated real-time and objective data. Front Psychol.

[79] Serra-Blasco M, Torres IJ, Vicent-Gil M, Goldberg X, Navarra-Ventura G, Aguilar E, Via E, Portella MJ, Figuereo I, Palao D, Lam RW, Cardoner N (2019). Discrepancy between objective and subjective cognition in major depressive disorder. Eur Neuropsychopharmacol.

