# Supplementary material for: Characterizing Longitudinal Patterns in Cognition, Mood, And Activity in Depression With 6-Week High-Frequency Wearable Assessment: Observational Study
Source: JMIR Ment Health. 2024 May 31;11:e46895. doi: 10.2196/46895 (PMC11179033; doi:10.2196/46895)
Supplement: Multimedia Appendix 1 [file mental_v11i1e46895_app1.docx]

| **Dependent variables** | **Number of observations** | **Model selection** | | | | | **Final model parameters** | | | | |
| --- | --- | --- | --- | --- | --- | --- | --- | --- | --- | --- | --- |
|  |  | **Trend fitted** | **AIC** | **BIC** | **Likelihood ratio** | **p-value** | **Predictors** | **Estimate** | **Standard error** | **t-value** | **p-value** |
| d-prime | 3251 | Intercept  Linear  Quadratic  Cubic | 6067.70  6041.16  5963.77  5954.46 | 6116.22  6083.77  6012.46  6009.24 | -  40.53  79.40  11.30 | -  <0.001  <0.001  <0.001 | Intercept  Time on task  (Time on task)^2^  (Time on task)^3^ | 1.17  0.003  -0.00004  1.8x10^-9^ | 0.10  0.0004  0.0000008  0.0000000 | 12.27  8.24  -4.81  3.36 | <0.001  <0.001  <0.001  <0.001 |
| Total mood  (log transformed) | 1094 | Intercept  Linear  Quadratic  Cubic | 441.15  437.76  439.51  441.07 | 471.14  472.75  479.50  486.15 | -  5.39  0.25  0.44 | -  0.02  0.61  0.51 | Intercept  Study day | 1.88  -0.0001 | 0.05  0.00005 | 36.82  -2.41 | <0.001  0.02 |
| Step count  (square root transformed) | 1131 | Intercept  Linear  Quadratic  Cubic | 10140.49  10138.96  10140.42  10141.48 | 10170.67  10174.17  10180.67  10186.76 | -  3.53  0.54  0.94 | -  0.06  0.46  0.33 | Intercept | 76.25 | 3.70 | 23.16 | <0.001 |
| Average daily Heart rate  (log transformed) | 1131 | Intercept  Linear  Quadratic  Cubic | -2312.89  -2311.23  -2310.46  -2309.32 | -2282.71  -2276.02  -2270.22  -2264.05 | -  0.34  1.23  0.86 | -  0.56  0.27  0.35 | Intercept | 4.40 | 0.015 | 287.70 | <0.001 |
